# Dual-function AAV gene therapy reverses late-stage Canavan disease pathology in mice

**DOI:** 10.3389/fnmol.2022.1061257

**Published:** 2022-12-08

**Authors:** Dominik Fröhlich, Elizabeth Kalotay, Georg von Jonquieres, Andre Bongers, Brendan Lee, Alexandra K. Suchowerska, Gary D. Housley, Matthias Klugmann

**Affiliations:** ^1^Translational Neuroscience Facility and Department of Physiology, School of Biomedical Sciences, University of New South Wales, Sydney, NSW, Australia; ^2^Biological Resources Imaging Laboratory, University of New South Wales, Sydney, NSW, Australia; ^3^Research Beyond Borders, Boehringer Ingelheim Pharma GmbH & Co. KG, Biberach an der Riss, Germany

**Keywords:** Canavan disease, leukodystrophy, gene therapy, adeno-associated virus, aspartoacylase, ASPA, NAT8L, NAA (N-acetyl-L-aspartate)

## Abstract

The leukodystrophy Canavan disease is a fatal white matter disorder caused by loss-of-function mutations of the aspartoacylase-encoding *ASPA* gene. There are no effective treatments available and experimental gene therapy trials have failed to provide sufficient amelioration from Canavan disease symptoms. Preclinical studies suggest that Canavan disease-like pathology can be addressed by either *ASPA* gene replacement therapy or by lowering the expression of the N-acetyl-L-aspartate synthesizing enzyme NAT8L. Both approaches individually prevent or even reverse pathological aspects in Canavan disease mice. Here, we combined both strategies and assessed whether intracranial adeno-associated virus-mediated gene delivery to a Canavan disease mouse model at 12 weeks allows for reversal of existing pathology. This was enabled by a single vector dual-function approach. *In vitro* and *in vivo* biopotency assessment revealed significant knockdown of neuronal *Nat8l* paired with robust ectopic aspartoacylase expression. Following nomination of the most efficient cassette designs, we performed proof-of-concept studies in post-symptomatic *Aspa*-null mice. Late-stage gene therapy resulted in a decrease of brain vacuoles and long-term reversal of all pathological hallmarks, including loss of body weight, locomotor impairments, elevated N-acetyl-L-aspartate levels, astrogliosis, and demyelination. These data suggest feasibility of a dual-function vector combination therapy, directed at replacing aspartoacylase with concomitantly suppressing N-acetyl-L-aspartate production, which holds potential to permanently alleviate Canavan disease symptoms and expands the therapeutic window towards a treatment option for adult subjects.

## Introduction

Leukodystrophies are inherited myelin diseases associated with substantial morbidity and mortality in children. Canavan disease (CD) is an autosomal-recessive leukodystrophy characterized by failure to attain developmental milestones, intellectual disability, seizures, and premature death. Brain pathology of CD patients shows progressive central nervous system (CNS) vacuolization, oedema, ventricle dilation, and oligodendrocyte loss.

CD is caused by loss of function mutations of the gene encoding aspartoacylase (ASPA; Kaul et al., 1993), a cytosolic oligodendroglial enzyme (Klugmann et al., 2003). Under normal conditions, ASPA hydrolyses N-acetyl-L-aspartate (NAA) into aspartate and acetate. The biochemical consequence of ASPA-deficiency is a build-up of NAA, and its derivative NAAG, in brain, blood and urine. NAA is the second most abundant free amino acid in the healthy CNS after glutamate and its role in the brain is still not completely understood. Increased NAA levels are used as a biomarker for the disease. The NAA metabolism is highly segmented with NAA production, catalyzed by the enzyme N-acetyltransferase 8 like (NAT8L) from Acetyl-CoA and aspartate, occurring in neurons, and NAA degradation taking place in oligodendrocytes following uptake through the sodium-dependent dicarboxylate transporter NaC3/SLC13A3. The etiology of CD has been thought to relate directly to cytotoxic and osmotic effects of excess NAA (Guo et al., 2015). This provided the rationale for lowering NAA *via* reducing its endogenous production. Proof-of-concept data were obtained from rescue experiments employing genetic deletion of *Nat8l* alleles in ASPA-deficient mouse lines in several laboratories (Guo et al., 2015; Maier et al., 2015; von Jonquieres et al., 2018). These findings were corroborated by *Nat8l* knockdown approaches (Bannerman et al., 2018; Hull et al., 2020).

In the absence of disease-modifying therapies, CD became the first neurogenetic brain disorder ever to be trialed by gene therapy, aiming at adding functional *ASPA* gene copies (Leone et al., 2000). While early trials showed minimal neurological benefit due to suboptimal vector technology (Leone et al., 2000, 2012), *ASPA* gene addition - using refined delivery systems - has been the subject of intensive research both in the clinic and in CD mouse models [for review see Lotun et al. (2021)]. In addition to the direct gene delivery approach, *ex vivo* gene therapy using patient derived induced pluripotent stem cells (iPSCs) has shown promise in CD mice (Feng et al., 2020; Chao et al., 2022).

In this study, we investigated potential added benefits of a dual-function vector approach aiming at combining *ASPA* gene addition and, at the same time, lowering *Nat8l* expression. ASPA-deficient lacZ-knock-in mice (AKO) accurately replicate CD-like pathology (Mersmann et al., 2011). Post-symptomatic treatment of aged AKO mice produced promising recovery from CD symptoms, including normalization of brain metabolites, reduced vacuolization and astrogliosis, improved motor behavior, and restored myelination. Moreover, intervention by optimized dual-function vectors might create a bigger therapeutic window not only for CD but also for other leukodystrophies.

## Materials and methods

### Animals

ASPA-deficient lacZ-knock-in mice (AKO) were used as a rodent CD model as described (Mersmann et al., 2011; von Jonquieres et al., 2018). All procedures were conducted in accordance with the Australian Code of Practice for the Care and Use of Animals for Scientific Purposes and were approved by the University of New South Wales Animal Care and Ethics Committee.

### Plasmid construction

All AAV plasmids used in this study were flanked by AAV2 inverted terminal repeats (ITR) and contained the woodchuck hepatitis virus post-transcriptional regulatory element (WPRE) and the bovine growth hormone polyadenylation signal (pA). RNAi target sequences were designed using the siRNA at Whitehead design tool.^1^ The shRNA sequences employed in this study were shNat8l.1: CACAGCTTCTCCCAGCCTGAA and shNat8l.2: GGTGGCCGCCCACAAGCTCTAT. The dual-function vectors contained either the *Nat8l* shRNAs under control of the RNA pol III U6 promoter or alternatively the corresponding target sequences designed as micro (mi)RNAs driven by the RNA pol II promoters CMV, CAG or Synapsin (SYN). The miRNA sequences used in this study were mirNat8l.1: ctcgactagggataacagggtaattgtttgaatgaggcttcagtactttacagaatcgttgcctgcacatcttggaaacacttgctgggattacttcgacttcttaacccaacagaaggctcgagaaggtatattgctgttgacagtgagcgCACAGCTTCTCCCAGCCTGAAtagtgaagccacagatgtaTTCAGGCTGGGAGAAGCTGTGtgcctactgcctcggacttcaaggggctagaattcgagcaattatcttgtttactaaaactgaataccttgctatctctttgatacatttttacaaagctgaattaaaatggtataaattaaatcactttttt and mirNat8l.2: ctcgactagggataacagggtaattgtttgaatgaggcttcagtactttacagaatcgttgcctgcacatcttggaaacacttgctgggattacttcgacttcttaacccaacagaaggctcgagaaggtatattgctgttgacagtgagcgGGTGGCCGCCCACAAGCTCTAtagtgaagccacagatgtaTAGAGCTTGTGGGCGGCCACCtgcctactgcctcggacttcaaggggctagaattcgagcaattatcttgtttactaaaactgaataccttgctatctctttgatacatttttacaaagctgaattaaaatggtataaattaaatcactttttt. The dual-function vectors also contained a modified cDNA of the human *ASPA* open reading frame driven by the 1.3 kb mouse MBP promoter (Lawlor et al., 2009). Modifications included codon-optimization of the human *ASPA* coding sequence for expression in mice and the addition of a Kozak sequence at the 5′-end of the cDNA. Codon adaptation was performed using the JCat algorithm available from TU Braunschweig (Grote et al., 2005). For ectopic ASPA expression only, codon-optimized or wildtype human *ASPA* were driven by the 1.3 kb MBP promoter.

### AAV vector production and stereotaxic injections

Packaging, purification, and titer quantification of AAV vectors (serotype cy5) was performed as described (von Jonquieres et al., 2013, 2016) with one deviation from the original protocol. We used polyethylenimine (PEI) transfection of HEK293 cells instead of calcium phosphate as described (Huang et al., 2013). For stereotaxic injections, anesthesia was induced in an induction chamber with 4% isoflurane in oxygen (0.8 l/min) and maintained at 1–2% isoflurane at 0.8 l/min oxygen flow rate during the surgery. Post-symptomatic mice were bilaterally injected at 12 or 22 weeks with 1 μl AAV solution containing 3 × 10^9^ viral genomes (vg) into the striatum (+0.7 mm AP, ±1.5 mm ML, −3.2 mm DV), thalamus (−1.5 mm AP, ±1.0 mm ML, −3.0 mm DV) and cerebellum (−5.6 mm AP, ±1.2 mm ML, −2.5 mm DV).

### Behavioral testing

The rotarod test was performed as described (Fröhlich et al., 2017). Mice were tested in a series of 3 trials per day. The rotation of the rod increased from 4 to 40 rpm over a period of 4 min. Cut-off time was 5 min. The individual performances were averaged over six trials performed on two consecutive days. For the hanging wire test, mice were placed on a wire 50 cm above the surface of soft bedding material. The latency to fall onto the bedding was recorded (cut-off time: 60 s).

### Magnetic resonance imaging (MRI) and ^1^H-MR spectroscopy (^1^H-MRS)

MR imaging was performed as described (Klugmann et al., 2022). General anesthesia was induced in an induction chamber with 4% isoflurane in oxygen (0.8 l/min) and maintained at 1–2% isoflurane at 0.8 l/min oxygen flow rate through a nose cone during the scanning procedure. Respiratory rate was monitored during the imaging procedures using a pressure sensitive pad. Animal body temperature was kept stable using a circulating water warming blanket. *In vivo* imaging was performed on a Bruker 9.4 T BioSpec Avance III 94/20 magnetic resonance microimaging system equipped with 15 mm internal diameter quadrature specimen volume coil. ^1^H-MR spectra from a 2 × 2 × 2 mm^3^ voxel in the thalamus were acquired at an echo time of 10 ms using a PRESS single voxel sequence as described (von Jonquieres et al., 2018).

### Immunocytochemistry

Cells were fixed in 4% paraformaldehyde (PFA) in PBS for 15 min. After 3 min permeabilization with 0.1% Triton X-100 in PBS and 30 min blocking with 4% horse serum in PBS, cells were incubated with primary rabbit anti-ASPA antibody [1:300; developed in house (Mersmann et al., 2011)] overnight at 4°C. Following 3 washes with PBS, cells were incubated at room temperature with goat anti-rabbit Alexa594 secondary antibody (1:300; Invitrogen). Nuclei were stained with the nuclear dye 4′,6-diamidino-2-phenylindole (DAPI).

### Immunohistochemistry

Immunohistochemistry was performed as described (Fröhlich et al., 2021). Brains were extracted, dissected along the midline and one hemisphere was fixed in 4% PFA, while tissue from the other hemisphere was snap frozen in liquid nitrogen for biochemical analyses. Following fixation, the hemisphere was cryo-protected in 30% sucrose solution, frozen at-80°C and cut into 40 μm sections on a cryostat. After permeabilization with 0.2% TritonX-100 in PBS (PBS-T), non-specific binding was blocked with 5% horse serum in PBS-T. Sections were incubated overnight at room temperature with the following primary antibodies in 5% horse serum in PBS-T: rabbit anti-ASPA [1:300; developed in house (Mersmann et al., 2011)], rabbit anti-NF200 (1:500; Sigma #N4142), rabbit anti-PLP clone aa3 (1:20; gift from Prof. J. Trotter, Mainz, Germany), mouse anti-GFAP (1:500; Cell Signaling #3670S). After washings with PBS-T, sections were incubated for 4 h at room temperature with appropriate Alexa fluorophore conjugated secondary antibodies (Invitrogen). FluoroMyelin^™^ Red (Thermo Fisher Scientific; #F34652) staining was performed in 5% horse serum in PBS-T according to the manufacturer’s instructions with the following deviations: concentration 1:250; incubation time 3 h. Following washing and staining of nuclei with DAPI, sections were mounted in ProLong^™^ Gold Antifade mounting media (Thermo Fisher Scientific; #P10144). Images were taken on a LSM710 confocal microscope (Zeiss). Image processing was performed using the ImageJ software including McMaster Biophotonics Facility (MBF) plugin collection.

### RNA isolation and qPCR

Quantitative RNA analysis was performed as described (Fröhlich et al., 2018). Cultured cells were directly lysed in the cell culture dish while brain tissue was homogenized first under liquid nitrogen using mortar and pestle. RNA was extracted using the RNeasy MiniKit following the manufacturer’s instructions (Qiagen #74106) including on-column DNase digest (RNase-Free DNase Set, Qiagen #79254). cDNA was synthesized from 500 ng total RNA using the High-Capacity cDNA Reverse Transcription Kit (Applied Biosystems #4368813) according to the manufacturer’s instructions. qPCR was performed on a StepOne Plus Real-Time PCR system (Applied Biosystems) using TaqMan assays (Applied Biosystems) specific for *Nat8l* (Mm01217216_m1) and *GusB* (Mm01197698_m1). To directly compare wildtype and codon-optimized *ASPA* mRNA levels, SYBR Green Mastermix (Applied Biosystems) together with primer pairs specific for *WPRE* and the housekeeper *Gapdh* were used. Comparative ΔΔCt values were determined relative to the housekeeping genes *GusB* and *Gapdh*, respectively.

### Western blotting

Western blotting was performed as previously described (Fröhlich et al., 2021). Cultured cells were directly lysed in the cell culture dish in lysis buffer (50 mM Tris-Cl, pH 7.4, 1 mM EDTA pH 8.0, 250 mM NaCl, and 1% Triton-X) containing a protease inhibitor cocktail (Roche Complete). Brain tissue was dissected, snap frozen in liquid nitrogen, and homogenized in liquid nitrogen using mortar and pestle. Brain homogenates were then lysed in lysis buffer containing protease inhibitors using a Branson 450 Digital Probe Sonifier at 10% sonication amplitude and protein concentration was determined by Bradford protein assay (Bio-Rad #5000006). 10 μg of protein were mixed with 5x sample buffer (15 g SDS, 15.6 ml 2 M Tris pH 6.8, 57.5 g glycerol, 16.6 ml β-mercaptoethanol), loaded onto a 10% acrylamide gel, separated by SDS-PAGE, and transferred onto PVDF membranes (Bio-Rad no. 162–0177). Membranes were blocked in 4% milk powder in 0.1% Tween / 1x PBS and probed with the following primary antibodies: rabbit anti-ASPA antibody [1:3,000; developed in house (Mersmann et al., 2011)], mouse anti-Actin clone C4 (1:10,000; Sigma-Aldrich #mab1501), mouse anti-CNP (1:3,000, Abcam #ab6319), rat anti-MBP (1:1,000; Abcam #ab7349), rat anti-PLP aa3 (1:200, gift from Prof. J. Trotter, Mainz, Germany), rabbit anti-GAPDH (1:4,000, Cell Signaling #2118S). After washing with 0.1% Tween / 1x PBS, HRP-conjugated secondary antibodies (Dianova) were applied in 4% milk powder in 0.1% Tween / 1x PBS. Membranes were developed with Clarity Western ECL substrate (BioRad #170–5,060) and imaged using the ChemidocMP system (BioRad).

### AAV vector genome quantification in tissue

To determine AAV vector genomes in the brains of AKO mice following gene therapy, brain tissue was first homogenized in liquid nitrogen using mortar and pestle. DNA was then extracted from 10 mg brain tissue using the DNeasy Blood and Tissue Kit (Qiagen) according to the manufacturer’s instructions. qPCR was performed on a StepOne Plus Real-Time PCR system (Applied Biosystems) using SYBR Green Mastermix (Applied Biosystems) together with primer pairs specific for *WPRE* and the housekeeper *Gapdh*. *WPRE* Ct values were normalized to *Gapdh* Ct values and AAV vector genomes were determined in relation to a *WPRE* standard curve.

### Statistics

Graphs and statistical analyses were performed with the Prism 8 software (GraphPad). One-way or Two-Way ANOVA with correction for multiple comparisons using the Bonferroni *post-hoc* test were used for statistical analysis as indicated.

## Results

### Vector design and *in vitro* biopotency of dual-function AAV constructs

**Figure 1:**
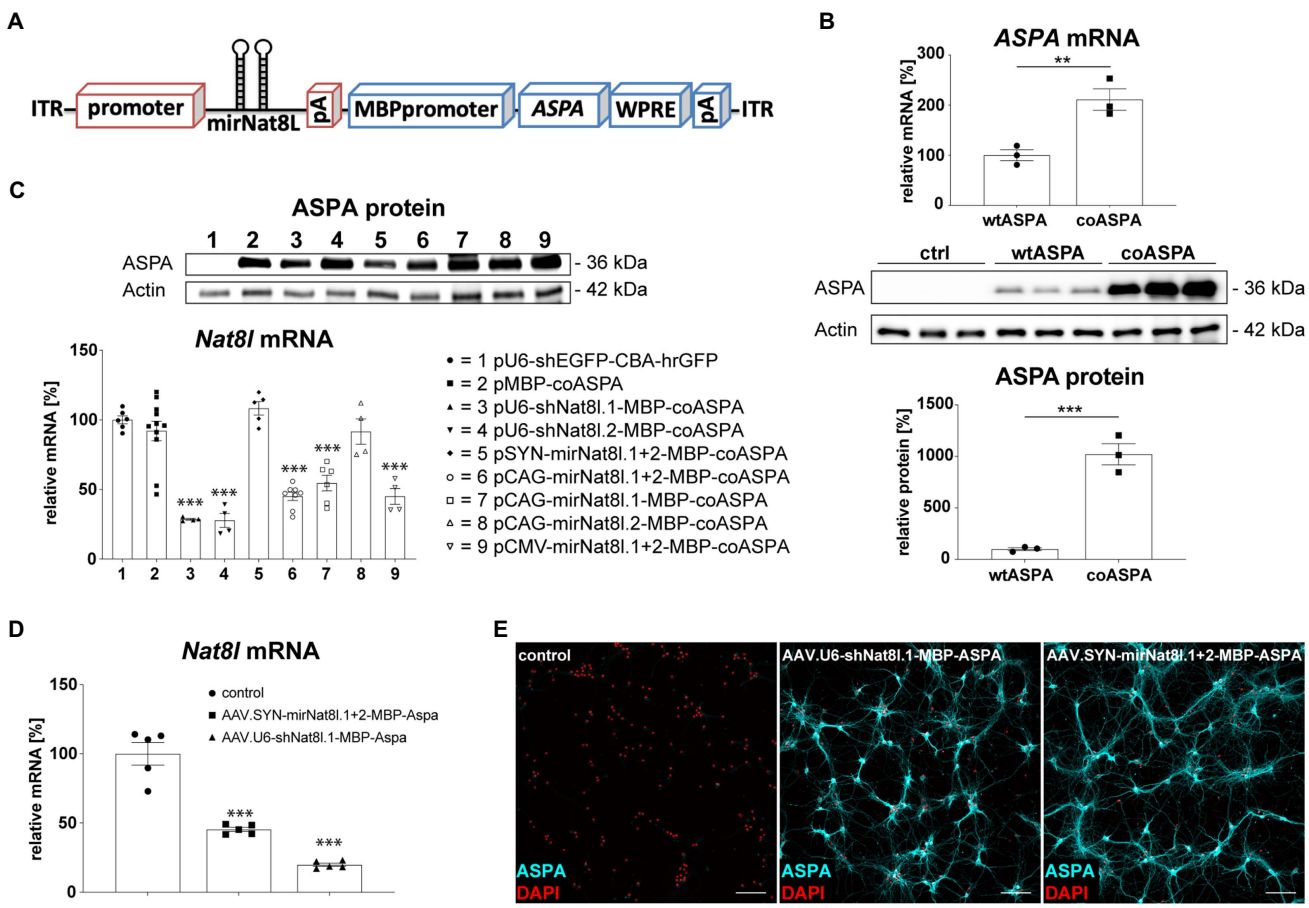
Vector design and *in vitro* biopotency of dual-function AAV vectors. **(A)** Design of dual-function AAV expression cassette. **(B)** Codon-optimization increases ASPA expression. HEK293 cells were transfected with equimolar amounts of wildtype ASPA *(wtASPA)* and codon-optimized *ASPA* (*coASPA*). **(B, top)** *ASPA* mRNA levels were determined by qPCR. ΔΔCt values normalized to the housekeeper *Gapdh* are displayed (*n* = 3). **(B, bottom)** Western blot depicting ectopic ASPA expression 3 days post transfection and densitometric quantification of ASPA normalized to the housekeeper Actin (*n* = 3). **(C)** Ectopic ASPA expression and *Nat8l* knockdown 3 days after transient transfection of the mouse neuroblastoma cell line N2A with different AAV plasmids. Western blot depicting ectopic ASPA expression 3 days post transfection. *Nat8l* levels were determined by qPCR using TaqMan probes specific for *Nat8l* and the housekeeper *GusB*. Displayed are ΔΔCt values normalized to untransfected cells (*n* = 4–11). **(D,E)** Analysis of dual-function AAV vectors in primary mouse neurons. Primary cortical **(D)** and hippocampal **(E)** neurons were transduced with 3 × 10^9^ vg of AAV.SYN-mirNat8l.1 + 2-MBP-ASPA, AAV.U6-shNat8l.1-MBP-ASPA, or AAV.U6-shEGFP-CBA-hrGFP (control) and analyzed 2 weeks post transduction. **(D)** *Nat8l* mRNA levels were determined *via* qPCR using TaqMan probes specific for *Nat8l* and *GusB*. Displayed are ΔΔCt values normalized to the housekeeping gene GusB and control transduced neurons (*n* = 5). **(E)** Immunocytochemistry for ASPA (cyan) and DAPI (red) in primary hippocampal neurons (scale bar: 100 μm). Data represent mean ± SEM. ^**^p < 0.01, ^***^p < 0.001; One-way ANOVA with Bonferroni *post-hoc* test.

The bi-cistronic AAV cassettes used in this study contained sequences against two different *Nat8l* mRNA targets or a combination of both, as well as a codon-optimized human *ASPA* (*coASPA*) open reading frame for improved expression in mice (Figure 1A). Transfection of HEK293 cells with *coASPA* or equimolar amounts of wildtype human *ASPA* (*wtASPA*) led to a 10-fold increase of ASPA protein expression following *coASPA* transfection (Figure 1B). Of note, *ASPA* mRNA levels were increased two-fold following ectopic expression of *coASPA* indicating higher transcription efficiency on top of increased mRNA translatability (Figure 1B).

We have demonstrated previously that a deletion of one *Nat8l* allele in AKO mice resulted in a therapeutic lowering of excess NAA and subsequent neurological improvements in AKO mice (von Jonquieres et al., 2018). This defined our success criteria of 50% *Nat8l* knockdown efficacy for RNAi candidates in this study. In an N2A mouse neuroblastoma cell-line transfection model, we used *Nat8l* shRNAs under control of the strong RNA pol III U6 promoter and separately used the corresponding target sequences designed as micro (mi)RNAs driven from RNA pol II promoters including CMV, CAG or Synapsin (SYN; Figure 1C). Effective silencing (75%) of endogenous *Nat8l* mRNA was observed in N2A cells for each of the shRNA targets (Figure 1C). The CMV and CAG promoters lead to somewhat weaker miRNA-dependent silencing (50–60%), whereas the SYN promoter was not efficient in N2A cells. mirNat8l.2 alone did not have an effect regardless of the promoter. Of note, the MBP promoter appeared to express the optimized *ASPA* cDNA efficiently in neuronal N2A cells (Figure 1C). A subset of the dual-function constructs was then packaged as serotype AAV.cy5 viral vectors with broad tropism for both neurons and oligodendrocytes (von Jonquieres et al., 2016, 2018). The shNat8l.1 vector achieved 70% mRNA silencing in primary mouse cortical neurons, while the SYN promoter-driven miRNA.1 + 2 vector still achieved 50% reduction (Figure 1D). In line with the N2A results, we detected robust ASPA immunoreactivity in primary mouse hippocampal neurons transduced with dual-function vectors suggesting that the recombinant MBP promoter is also active in cultured neurons (Figure 1E). This finding was confirmed in primary cortical neurons (not shown).

### Biopotency of dual-function AAV vectors *in vivo*

**Figure 2:**
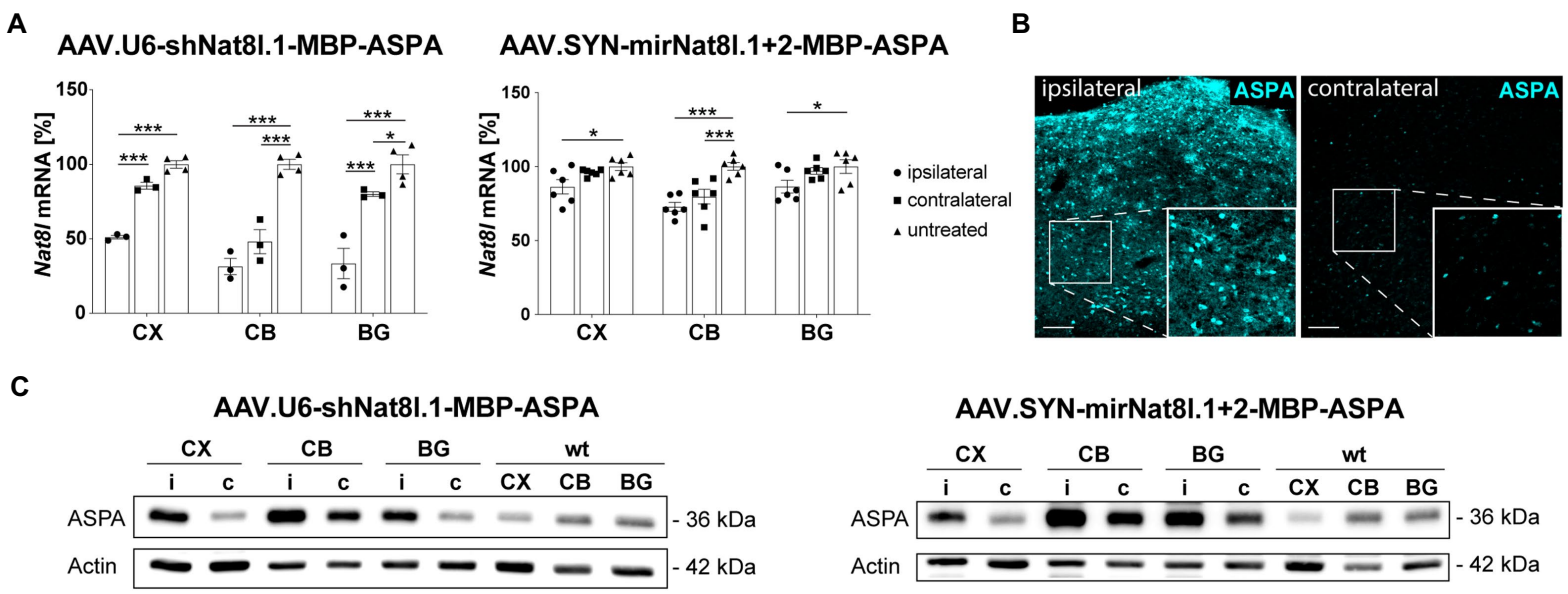
*In vivo* test of dual-function AAV vectors. AAV.U6-shNat8l.1-MBP-ASPA or AAV.SYN-mirNat8l.1 + 2-MBP-ASPA were injected unilaterally into striatum, thalamus, and cerebellum (3 × 10^9^ vg per site) of heterozygous AKO mice. *Nat8l* knockdown **(A)** and ASPA expression **(B,C)** in the injected (ipsilateral) hemisphere were determined 4 weeks post injection and compared to the contralateral side as well as to untreated controls. **(A)** *Nat8l* mRNA levels in the cortex (CX), cerebellum (CB) and basal ganglia (BG; including striatum and thalamus) were determined employing qPCR with TaqMan probes specific for *Nat8l* and the housekeeping gene *GusB* (*n* = 3 for AAV.U6-shNat8l.1-MBP-ASPA and *n* = 6 for AAV.SYN-mirNat8l.1 + 2-MBP-ASPA). Data represent mean ± SEM. ^*^p < 0.05, ^***^p < 0.001; Two-way ANOVA with Bonferroni *post-hoc* test. **(B)** Immunohistochemistry for ASPA in the thalamus of AAV.SYN-mirNat8l.1 + 2-MBP-ASPA treated, heterozygous AKO mice (scale bar: 100 μm). **(C)** Western blot depicting ASPA expression in the treated, ipsilateral (i) hemisphere and the untreated, contralateral (c) side of heterozygous AKO mice in comparison to untreated wt control mice.

We aimed at corroborating *in vivo* the results obtained with dual-function shRNA and miRNA vectors in primary neurons. For this purpose, we delivered the vectors *via* unilateral multi-site injections to the striatum, thalamus, and cerebellum of adult heterozygous AKO mice and analyzed dissected brain subregions 4 weeks post injection. Three sample groups were compared including the tissues from the injected hemisphere (ipsilateral), the non-injected hemisphere (contralateral) and control tissue from a naïve mouse. Analyses included qPCR for *Nat8l* mRNA levels and ASPA protein detection by immunoblot or immunohistochemistry (Figure 2). We detected strong *Nat8l* mRNA silencing in all regions for the shRNA vector tested (Figure 2A). A more moderate yet significant knockdown was achieved using the miRNA vector (Figure 2A). Both vectors led to substantially increased ASPA protein expression compared to endogenous levels (Figures 2B,C).

### Gene therapy in 22-week-old AKO mice

**Figure 3:**
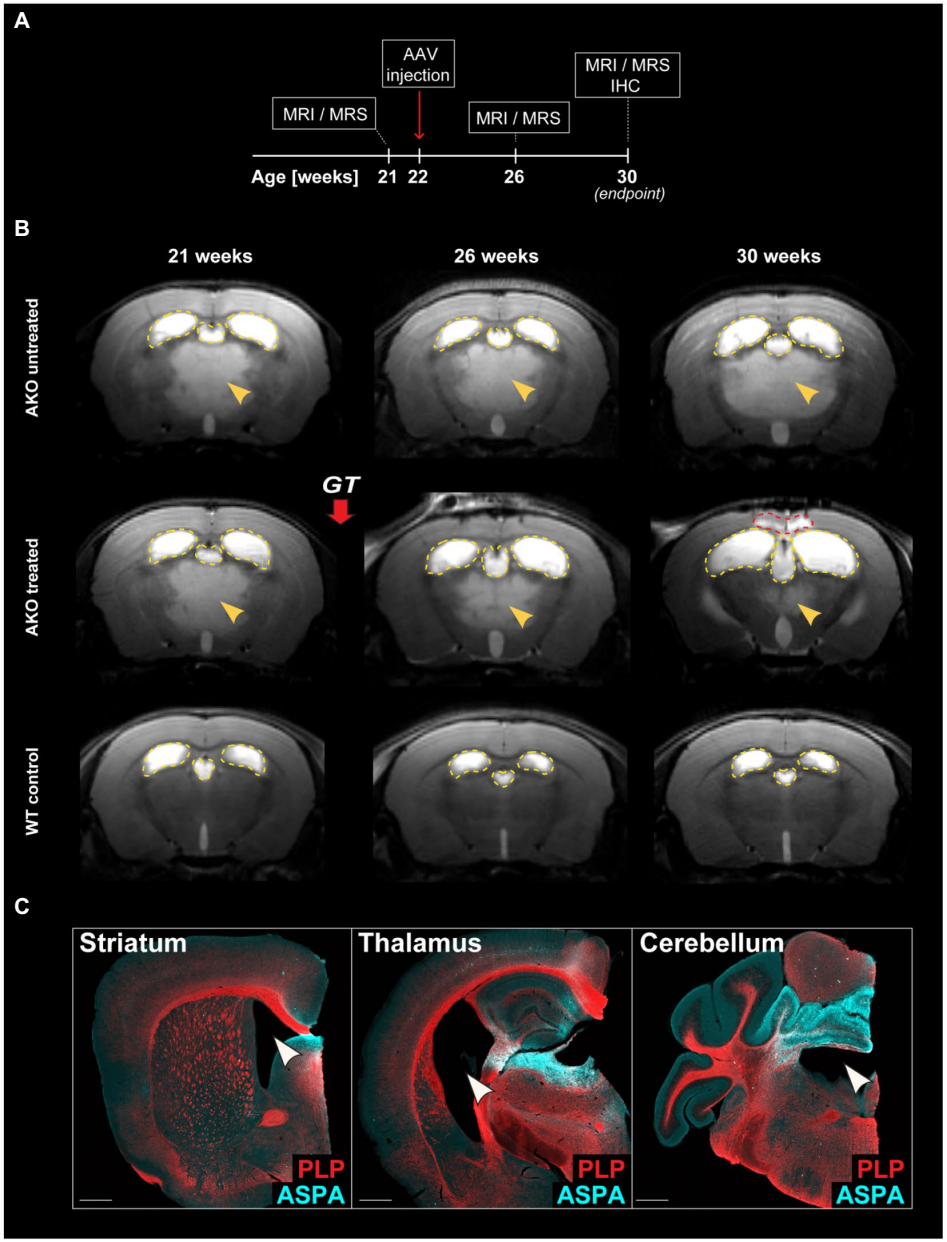
Dual function gene therapy in 22-week-old AKO mice with end-stage Canavan pathology reverses brain vacuolization but worsens ventricle dilation and neurological aspects of the disease. **(A)** Schematic depicting the study design for the treatment of AKO mice with end-stage Canavan pathology. **(B)** AAV.U6-shNat8l.1-MBP-coASPA vectors were injected bilaterally into striatum, thalamus, and cerebellum (3 × 10^9^ vg per site) of 22-week-old AKO mice. Mice were imaged before as well as 4-and 8-weeks post gene therapy (GT). Representative T2-weighted brain MRI images are displayed. Yellow arrowheads indicate T2 hyperintensities reflecting spongiform vacuolization. Yellow dashed lines outline the lateral and third ventricles. Red dashed line hightlights the area of water ingress into the frontal cortex of 30-week-old treated AKO mice. **(C)** Coronal brain sections from the AAV injected regions (striatum, thalamus, and cerebellum) were immunolabelled 8 weeks after gene therapy. Ectopic ASPA expression (cyan) and the myelin marker proteolipid protein (PLP; red) are shown. Arrowheads indicate worsened ventricle dilation in treated AKO mice. Scale bars: 500 μm.

**Figure 4:**
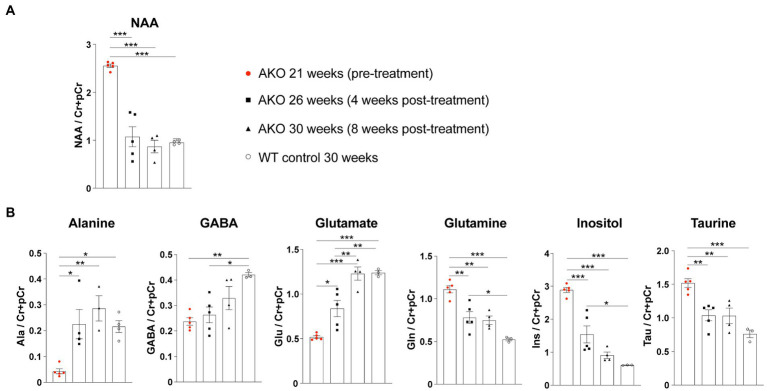
Dual function gene therapy in 22-week-old AKO mice with end-stage Canavan pathology normalizes brain metabolite levels. AAV.U6-shNat8l.1-MBP-coASPA vectors were injected bilaterally into striatum, thalamus, and cerebellum (3 × 10^9^ vg per site) of 22-week-old AKO mice. ^1^H-MRS was performed before as well as 4-and 8-weeks post-treatment. **(A)** Quantification of brain N-acetyl-L-aspartate (NAA) levels normalized to creatine and phosphocreatine (Cr + pCr) in the same group of AKO mice before and after treatment and in wildtype (WT) control mice (*n* = 4–5). **(B)** Quantification of the metabolites alanine (Ala), gamma aminobutyric acid (GABA), glutamate (Glu), glutamine (Gln), inositol (Ins), and taurine (Tau) normalized to creatine and phosphocreatine (Cr + pCr) in the brain of AKO mice before and after treatment and in WT control mice (*n* = 3–5). Data represent mean ± SEM. *p < 0.05, **p < 0.01 ***p < 0.001; One-way ANOVA with Bonferroni *post-hoc* test.

We have reported long-term therapeutic benefits following AAV.MBP-wtASPA delivery to symptomatic CD mice (AKO) at 4 weeks of age and the potential to reverse the brain pathology present at the time of intervention (von Jonquieres et al., 2018). In the current study, we employed our optimized vector designs and aimed to identify the latest time point of intervention for therapeutic effects and, at least partial, functional restoration. In a pilot study, we attempted to treat AKO mice with end-stage CD pathology at 22 weeks of age by multi-site delivery of AAV.U6-shNat8l.1-MBP-coASPA (Figures 3A). This treatment resulted in decreased thalamic vacuolization (Figure 3B) and normalization of brain metabolites including NAA (Figure 4) at 30 weeks of age. However, we observed water ingress into frontal cortex regions (Figure 3B), ventricle dilation (Figures 3B,C), and deteriorated neurological function, including motor seizures, resulting in euthanasia or death of the AAV-treated AKO mice compared with AKO controls. This finding warranted an earlier time point of intervention.

### Gene therapy in 12-week-old AKO mice

**Figure 5:**
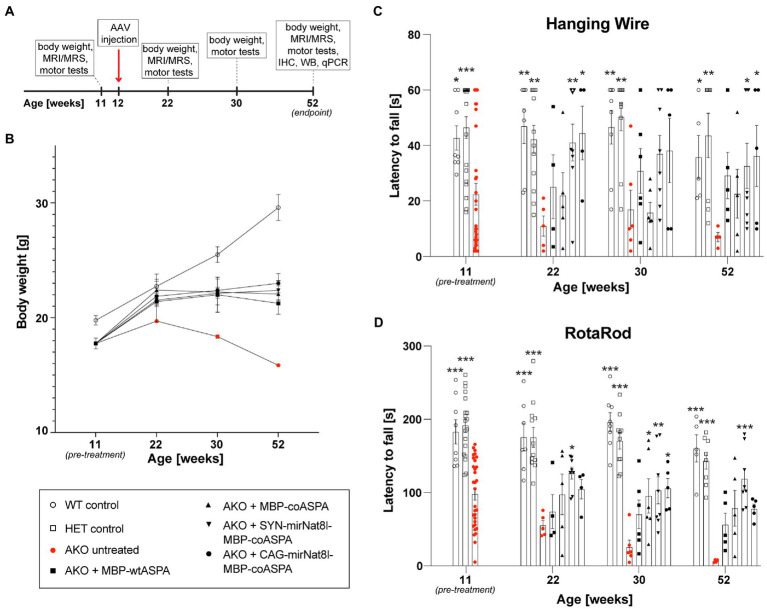
Dual-function AAV vectors improve motor function and stop age related weight loss in post-symptomatic (12-week-old) AKO mice. **(A)** Schematic depicting the study design for the treatment of AKO mice with advanced-stage Canavan pathology (IHC = immunohistochemistry, WB = western blotting, qPCR = quantitative real-time PCR). **(B-D)** AAV vectors were injected bilaterally into striatum, thalamus, and cerebellum (3 × 10^9^ vg per site) of 12-week-old AKO mice. Body weight **(B)** and motor behavior **(C,D)** were assessed before the treatment as well as 10-, 18-, and 40-weeks post-treatment using the Hanging Wire **(C)** and Rota Rod **(D)** tests (*n* = 4–8). Data represent mean ± SEM. ^*^p < 0.05, ^**^p < 0.01 ^***^p < 0.001; Two-way ANOVA with Bonferroni *post-hoc* test. Significance is displayed in comparison to AKO untreated.

We then employed the dual-function vector approach in 12-week-old AKO mice with advanced-stage CD-like symptoms by bilateral multi-site gene delivery. Mice were monitored for body weight and subjected to behavioral testing (Hanging wire and Rotarod tests) longitudinally before the treatment commenced as well as at 10-, 18-and 40-weeks post treatment (Figure 5A). The treatment groups included AKO + AAV.SYN-mirNat8l.1 + 2-MBP-coASPA, AKO + AAV.CAG-mirNat8l.1-MBP-coASPA, AKO + AAV.MBP-coASPA, AKO + AAV.MBP-wtASPA, AKO untreated, and heterozygous AKO and wildtype controls. We opted for miRNA over shRNA vectors as miRNAs are generally associated with less off-target and side effects compared to shRNAs (Grimm et al., 2006).

Treatment with all vectors resulted in reduced weight loss (Figure 5B) and improved motor behavior (Figures 5C,D) compared to untreated AKO mice. Overall, there appeared to be a slight advantage of the dual-function vectors AAV.SYN-mirNat8l.1 + 2-MBP-coASPA and AAV.CAG-mirNat8l.1-MBP-coASPA over *ASPA* gene addition alone.

### Reversed neuroanatomy and metabolite levels following CD gene therapy

**Figure 6:**
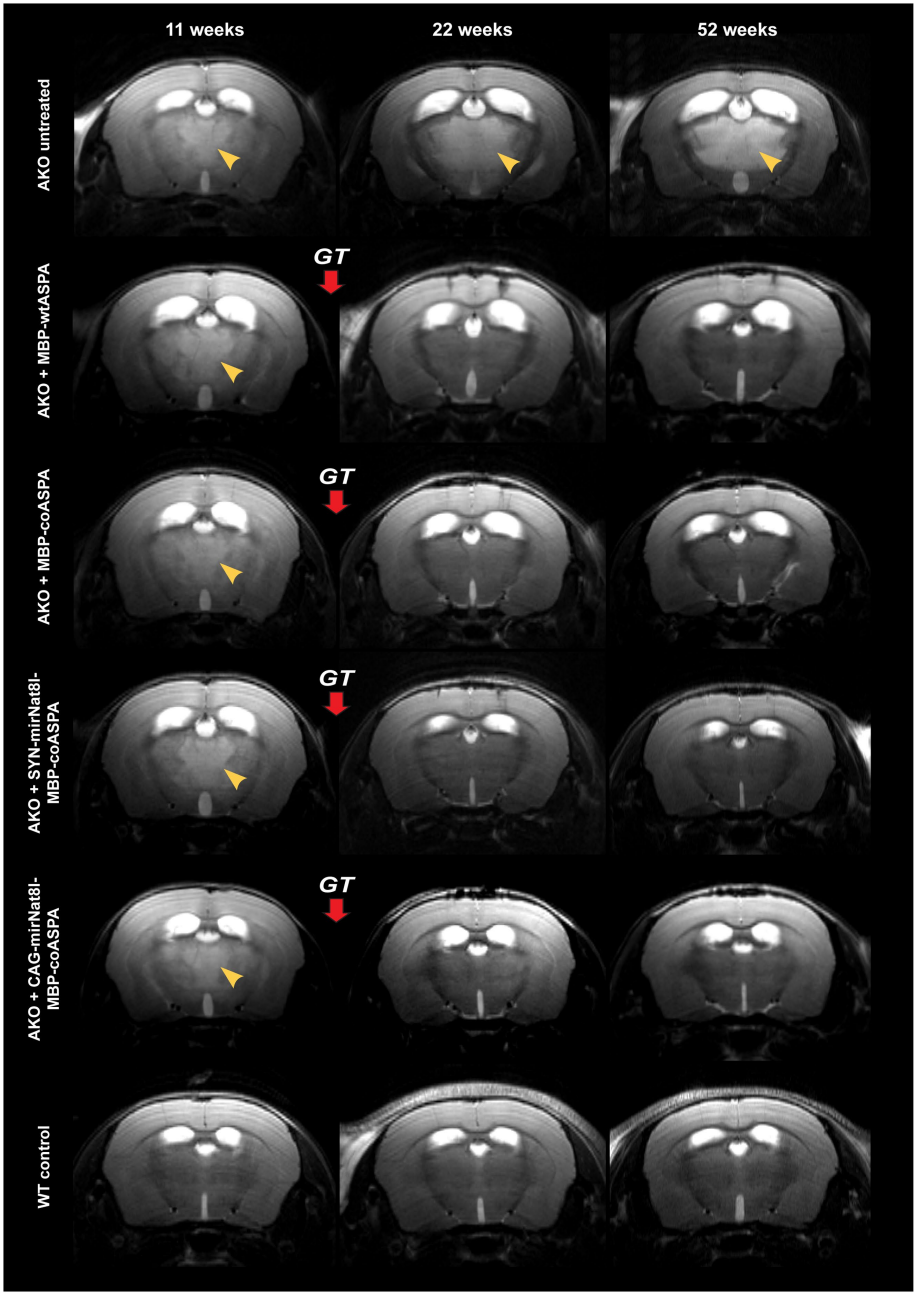
Treatment with novel gene therapy vectors reverses brain vacuolization and ventricle dilation in aged AKO mice. AAV vectors were injected bilaterally into striatum, thalamus, and cerebellum (3 × 10^9^ vg per site) of 12-week-old AKO mice. The same mice were imaged before as well as 10-and 40-weeks post gene therapy (GT). Representative T2-weighted brain MRI images are displayed. Yellow arrowheads indicate T2 hyperintensities reflecting progressive spongiform vacuolization.

We used non-invasive high-resolution *in vivo* Magnetic resonance imaging (MRI) and ^1^H-Magnetic resonance spectroscopy (^1^H-MRS) for assessment of the neuroanatomy and the baseline levels of the toxicity-inducing biomarker NAA prior to treatment as well as longitudinally at 10-, 18-and 40-weeks post treatment. MRI images obtained prior to vector delivery show the severe brain pathology present in 11-week-old AKO mice including T2 hyperintensities indicative of spongiform vacuolization (Figure 6, yellow arrowheads) and enlarged ventricles (Figure 6). This pathology worsens in untreated AKO mice over time (Figure 6, top row), while treatment with all vectors tested in this study completely reversed brain vacuolization (Figure 6; rows 2–5). Ventricle dilation was also reduced in all treatment groups; however, the effect appears to be more pronounced in AKO mice treated with dual-function vectors (Figure 6; rows 4 and 5).

**Figure 7:**
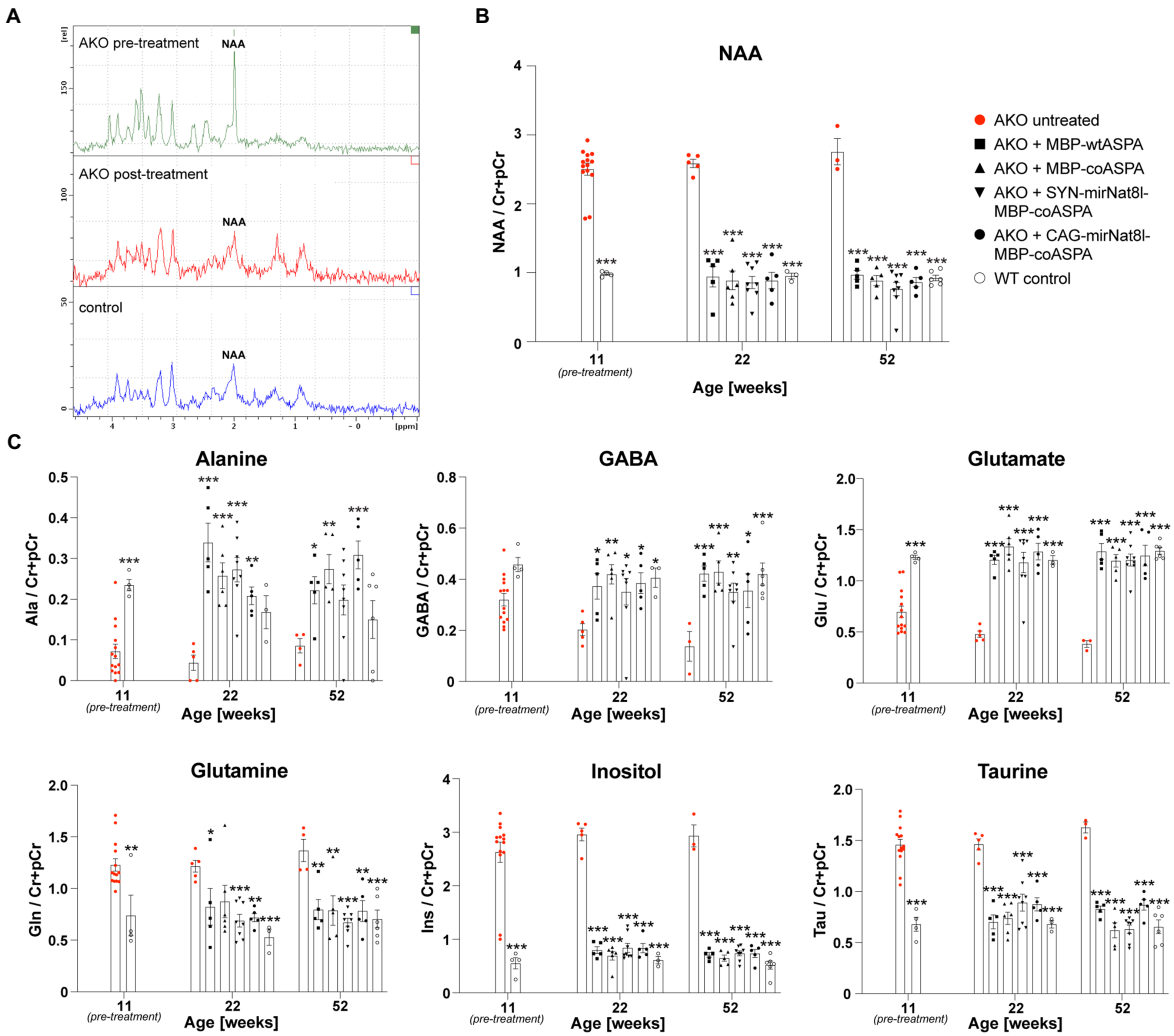
Restored brain metabolite levels in post-symptomatic AKO mice following AAV treatment. AAV vectors were injected bilaterally into striatum, thalamus, and cerebellum (3 × 10^9^ vg per site) of 12-week-old AKO mice. **(A)** Representative ^1^H-MRS spectra from AKO pre-treatment (top), post-treatment (AAV.SYN-mirNat8l.1 + 3-MBP-Aspa, middle) and from a wildtype control mouse (bottom). **(B)** Quantification of brain N-acetyl-L-aspartate (NAA) levels normalized to creatine and phosphocreatine (Cr + pCr) in the same group of AKO mice before gene therapy as well as 10-and 40-weeks post treatment and in wildtype (WT) control mice (*n* = 3–8). **(C)** Quantification of the metabolites alanine (Ala), gamma aminobutyric acid (GABA), glutamate (Glu), glutamine (Gln), inositol (Ins), and taurine (tau) normalized to Cr + pCr in the brain of AKO mice before as well as 10-and 40-weeks post treatment and in WT controls (*n* = 3–8). Data represent mean ± SEM. ^*^p < 0.05, ^**^p < 0.01 ^***^p < 0.001; Two-way ANOVA with Bonferroni *post-hoc* test. Significance is displayed in comparison to AKO untreated.

The supraphysiological NAA levels detected in mutants at the beginning of the treatment could be readily normalized to control levels (Figures 7A,B). Even 40 weeks post gene therapy the brain NAA levels remained on wildtype levels (Figure 7B). All tested vectors lowered supraphysiological NAA levels to the same extent with no obvious advantage of dual-function vectors over ectopic ASPA expression alone (Figure 7B). These data suggest that differences in efficacy of the vectors are masked by a ceiling effect at the selected dose. In addition to NAA, we measured other brain metabolites using *in vivo* ^1^H-MRS before as well as 10-and 40-weeks post treatment (Figure 7C). These metabolites included alanine, gamma-aminobutyric acid (GABA), glutamate, glutamine, inositol, and taurine. Alanine, GABA, and glutamate levels were reduced in the brains of AKO mice compared to healthy controls, while glutamine, inositol, and taurine levels were elevated. Following gene therapy, restoration to normal wildtype levels was observed across all brain metabolites examined, which was sustained until at least 40 weeks post gene therapy (Figure 7C).

### CD gene therapy restores myelin protein expression

**Figure 8:**
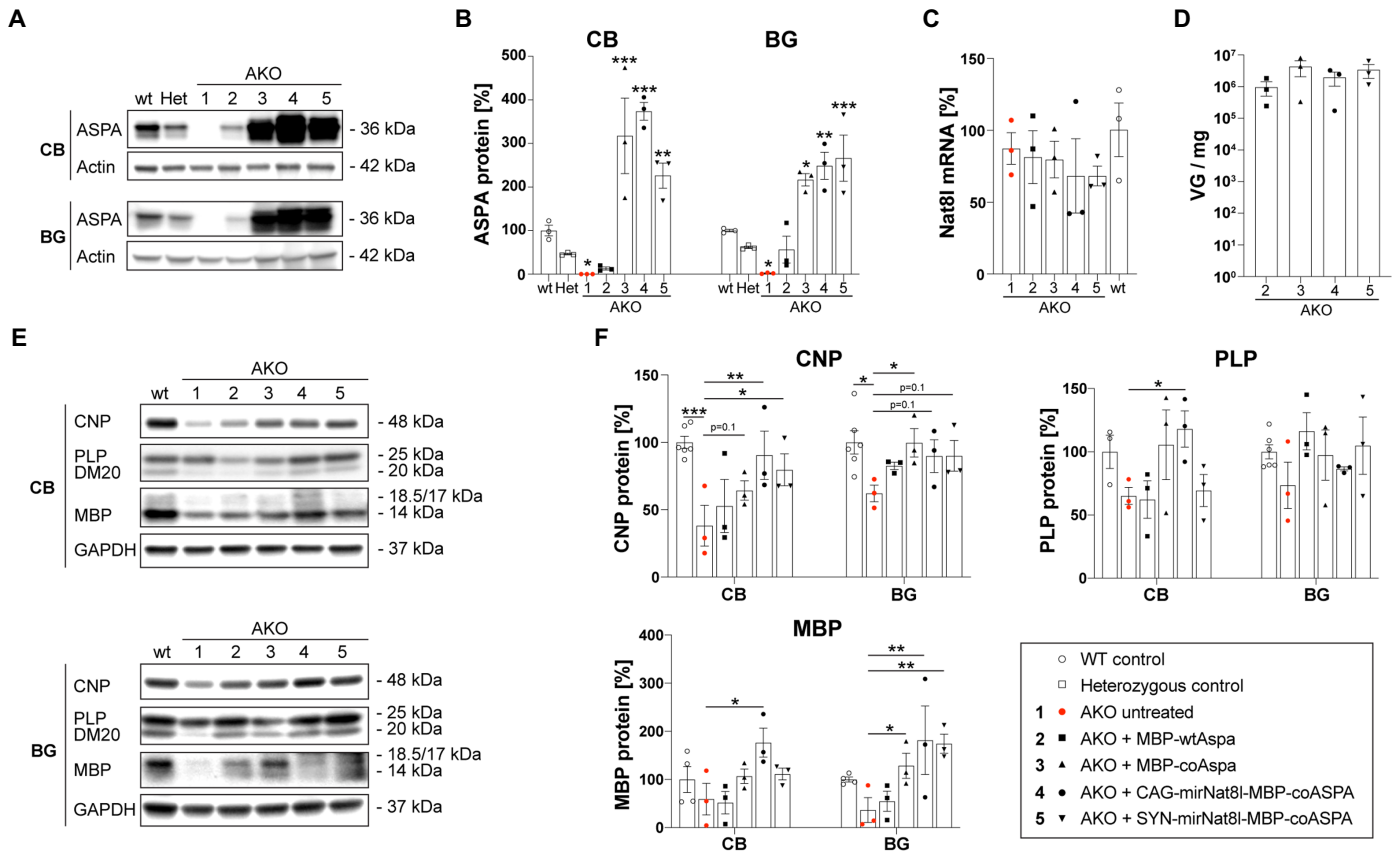
Restored myelin protein expression in AKO mice following dual-function gene therapy. AAV vectors were injected bilaterally into striatum, thalamus, and cerebellum (3 × 10^9^ vg per site) of 12-week-old post-symptomatic AKO mice. **(A)** Western blot showing ASPA expression in the cerebellum (CB) and basal ganglia (BG; including striatum and thalamus) of one-year-old mice 40 weeks post gene therapy. **(B)** Densitometric quantification of ASPA protein levels normalized to the housekeeper Actin and wildtype controls (*n* = 3). **(C)** *Nat8l* mRNA expression levels in the brain of one-year-old mice 40 weeks post gene therapy. Displayed are ΔΔCt values normalized to the housekeeping gene GusB and wildtype controls (*n* = 3). **(D)** AAV vector load in the brain of one-year-old mice 40 weeks post gene therapy measured as viral genomes per mg of brain tissue (*n* = 3). **(E)** Western blot depicting expression levels of the mayor myelin proteins CNP, PLP/DM20 and MBP as well as the housekeeping protein GAPDH in the CB and BG region of one-year-old mice 40 weeks post gene therapy (*n* = 3). **(F)** Densitometric quantification of the myelin proteins CNP, PLP and MBP normalized to the housekeeper GAPDH and wildtype controls (*n* = 3). Data represent mean ± SEM. ^*^p < 0.05,^**^p < 0.01, ^***^p < 0.001; Two-way ANOVA with Bonferroni *post-hoc* test.

To address whether late-stage CD gene therapy has the potential to restore myelination in AKO mice, we examined brain tissue of one-year-old mice at 40 weeks post gene therapy. Western blotting revealed strong ASPA expression in the cerebellum (CB) and basal ganglia (BG; including striatum and thalamus) 40 weeks post treatment (Figures 8A,B). The ASPA protein levels achieved by codon-optimization were overall about 10-fold higher compared to the non-optimized cDNA. *Nat8l* mRNA expression in the brain of dual-function vector-treated AKO mice was reduced yet did not reach statistical significance (Figure 8C). This might be due to dilution effects of surrounding brain tissue not affected by RNAi. AAV transduction efficiency in the brains of AKO mice 40 weeks post therapy was comparable between treatment groups with no significant differences detected (Figure 8D). Expression of the major myelin proteins 2′,3’-Cyclic-nucleotide 3′-phosphodiesterase (CNP), Proteolipid protein (PLP) and its splice variant DM20, and Myelin basic protein (MBP) were normalized following gene therapy treatment (Figures 8E,F).

### CD gene therapy normalizes brain anatomy and myelination

**Figure 9:**
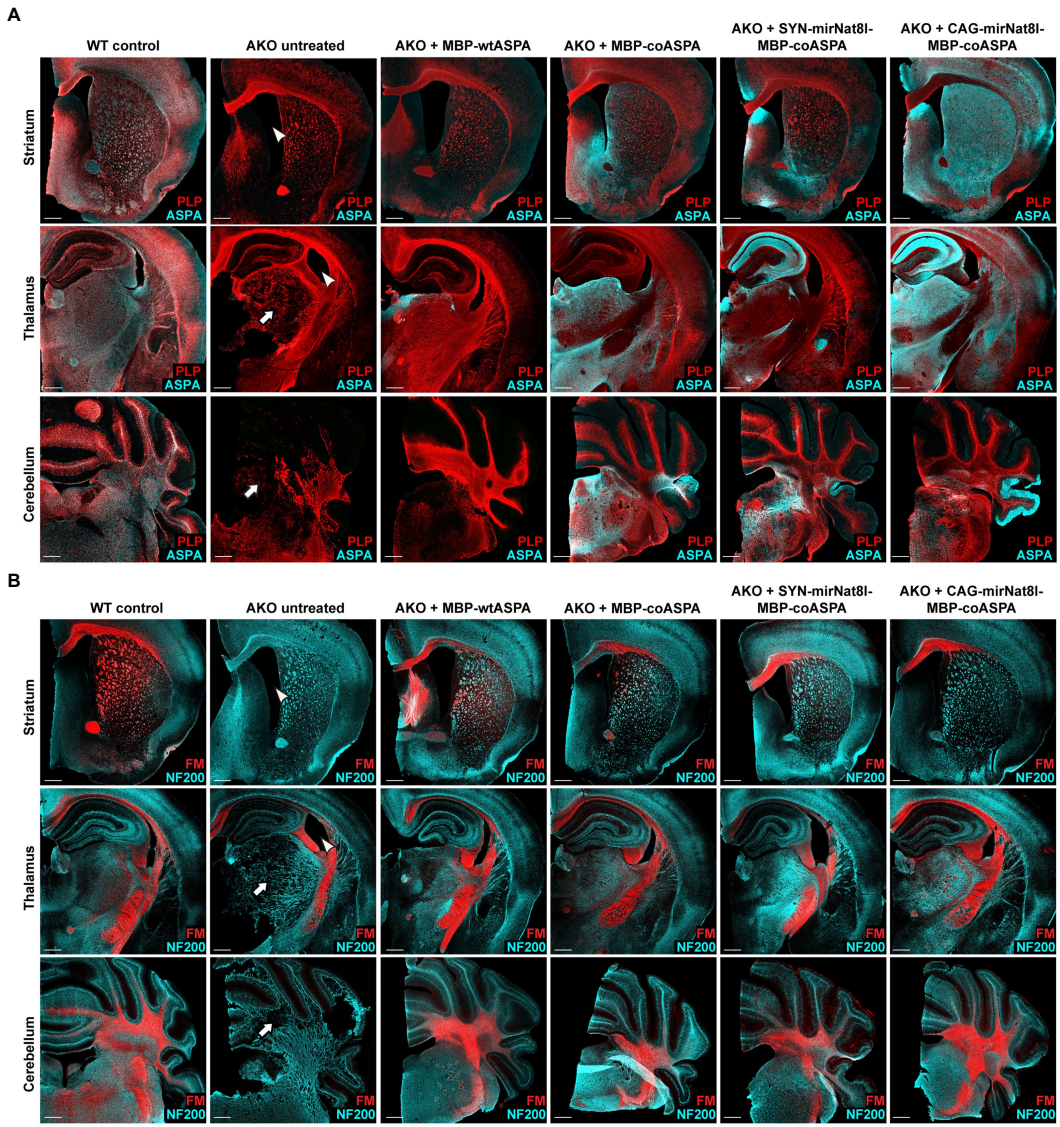
Treatment with novel CD gene therapy vectors restores ASPA expression and improves myelination in AKO mice. AAV vectors were injected bilaterally into striatum, thalamus, and cerebellum (3 × 10^9^ vg per site) of 12-week-old post-symptomatic AKO mice. Coronal brain sections from the AAV injected regions (striatum, thalamus and cerebellum) were immunolabelled 40 weeks after gene therapy. **(A)** Immunohistochemistry for ASPA (cyan) and PLP (red) to visualize vector spread and myelin integrity, respectively. Untreated AKO mice display enlarged ventricles (arrowheads) and spongiform vacuolization (arrows), both of which significantly improved following gene therapy. **(B)** Co-labelling with the neuronal marker NF200 (cyan) and FluoroMyelin Red (FM; red) showing improved myelination following AAV treatment. Scale bars: 500 μm.

Coronal brain sections taken from the striatum, thalamus, and cerebellum of mice 40 weeks post gene therapy were immunohistochemically labelled to qualitatively investigate AAV vector spread, and to further assess the efficacy of each treatment in normalizing CD brain pathology. Immunostaining verified the absence of ASPA in untreated AKO mice and successful transduction in all treated mice, with widespread and long-term AAV-mediated ASPA expression (Figure 9A). Untreated AKO displayed enlarged ventricles, spongiform vacuolization, and extensive demyelination, evidenced by a reduction in PLP immunofluorescence (Figure 9A) and FluoroMyelin red staining (Figure 9B). Despite the comparatively low levels of ASPA immunoreactivity detected in MBP-wtASPA treated brains, all treatments successfully reversed brain tissue vacuolization and improved myelination (Figures 9A,B).

### Reduced astrogliosis following CD gene therapy

**Figure 10:**
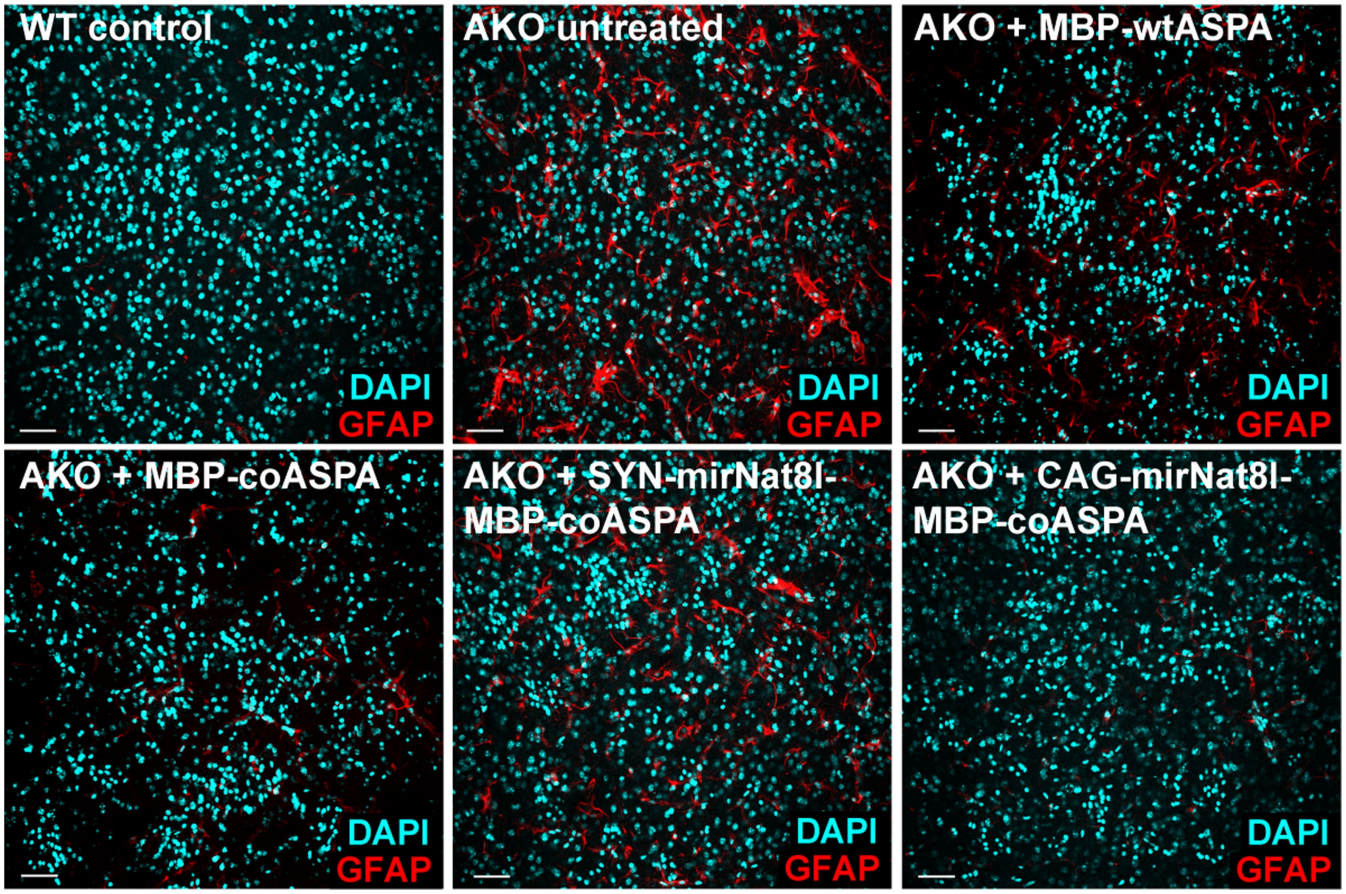
Astrogliosis in AKO mice is attenuated following treatment with novel CD gene therapy vectors. Immunohistochemical labelling of GFAP (astrocytes; red) and DAPI (nuclei; cyan) in the striatum of 52-week-old mice showing astrogliosis in untreated AKO mice, which markedly improved in all treatment groups 40 weeks post gene therapy. Scale bars: 50 μm.

Astrogliosis is a hallmark of CD. Consistent with that, AKO mice exhibit a marked increase in GFAP immunofluorescence compared to wildtype controls (von Jonquieres et al., 2018), which we confirmed in this study (Figure 10). Astrogliosis was significantly reduced in all treatment groups, with the strongest improvements following MBP-coASPA and CAG-mirNat8l-MBP-coASPA treatment.

## Discussion

*ASPA* gene addition or *Nat8l* suppression are beneficial in CD models, when targeted separately [for review see Lotun et al. (2021)]. In this study, we hypothesized that combining both concepts would have additive effects. We reasoned that for optimizing the outcome a single-vector design is warranted. This was enabled by a dual-function AAV cassette design for simultaneous ASPA expression and RNAi-based knockdown of endogenous *Nat8l* mRNA.

The concept of employing AAV-gene delivery for silencing a toxic allele with concomitant augmentation of a functional copy of the same gene has been pursued to target gain-of-function mutations in metabolic disorders (Mueller et al., 2012). Here, we adapted the technology for CD by targeting different genes for augmentation (*ASPA*) and silencing (*Nat8l*).

RNA interference alone using AAV.U6-shRNA to silence *Nat8l* in the ASPA^Nur7^ CD mouse model has been reported to partially prevent CD pathology when delivered pre-emptively (Bannerman et al., 2018). However, this neonatal treatment strategy is translationally of limited relevance given that the developmental maturity of the murine brain at birth is equivalent to that at the end of the first human trimester and that patients are more likely to be treated as infants, after the disease has been diagnosed and symptoms have already manifested. In adult wildtype brains, we observed shRNA-mediated *Nat8l* silencing efficiencies of more than 50%, meeting our criterion that was defined by the observed phenotypic normalization of CD symptoms in AKO mice with only one *Nat8l* allele (von Jonquieres et al., 2018). However, in the light of reported safety concerns due to AAV.shRNA delivery (Grimm et al., 2006), we compromised on *Nat8l* silencing efficacy and opted for a miRNA design, resulting in a lower, 20–30% knockdown (Figure 2A). In contrast to wildtype animals, the observed degree of *Nat8l* silencing did not reach statistical significance in AKO mice 40 weeks post transduction (Figure 8C). Following administration to aged AKO mice, we observed substantial improvements in all parameters examined for all different vector designs. The single-dose study design in this study may have precluded a better differentiation of the *in vivo* biopotency across the vectors. This may be addressed by a dose escalation study. Despite an assay-dependent trend towards some superiority of the dual-function design, the treatment effects were largely attributable to the *ASPA* gene augmentation, which was designed into all vector cassettes in this study. Following gene therapy in AKO, we observed ASPA protein levels between 15 and 380% of wildtype controls (Figure 8B). At the lower range of this large treatment window, we still observed substantial neurological, biochemical, and pathological ameliorations. The upper end of this spectrum was both tolerated and beneficial. This is an important learning of translational relevance. We note that compared with the naïve open reading frame, codon-optimization of the human *ASPA* cDNA resulted in dramatically improved DNA/protein ratio, similar to the report by Gessler et al. (2017). To further improve this substantial therapeutic outcome and to achieve a complete cure of CD in the future, it might be necessary to supplement intracranial delivery of dual-function vectors with systemic delivery, as some of the remaining symptoms such as reduced body size and weight might be a consequence of ASPA deficiency in the periphery (Ahmed et al., 2016; von Jonquieres et al., 2018).

AAV vectors were delivered directly to the brain by intracranial injections, an administration route that has been demonstrated to be safe in a clinical CD trial (Leone et al., 2012). We used the AAV serotype cy5, a variant of AAV7 with low immunogenicity and the potential to be used as a clinical gene therapy vector (Gao et al., 2002).

Early CD gene therapy approaches utilizing first generation AAV vectors in models (Matalon et al., 2003; Klugmann et al., 2005) or the clinic (Leone et al., 2012; Ahmed et al., 2013; von Jonquieres et al., 2018) have resulted in incomplete therapeutic outcomes. Preclinical AAV-mediated shRNA delivery against *Nat8l* was given in a prophylactic setting before CD-like symptoms were present, thereby limiting the translational relevance (Bannerman et al., 2018).

Pathological reversal upon preclinical *ASPA* gene delivery to two different CD models at 3 weeks or 6 weeks has been reported (Ahmed et al., 2013; Francis et al., 2021). In this study, we attempted to push the time point of intervention to the maximum. We reasoned that a dual-function approach is warranted for a successful proof-of-concept study in 12-week-old AKO mice, when all CD-like symptoms have fully manifested. Strikingly, treatment with the novel gene therapy vectors did not only stop disease progression but reversed the majority of the already developed pathophysiology. Another arm of this study aimed at gene delivery to AKO mice at 22 weeks, which resulted in remission of thalamic edema and normalization of neurometabolic markers, including NAA, but also an anatomical redistribution of brain water (Figure 3B), concomitant neurological deterioration and death. AAV.U6-shNat8l.1-MBP-ASPA was selected for this very challenging late time point of intervention because this cassette design showed the strongest *Nat8l* knock-down. The neurological deterioration of this group suggests that 22 weeks, in this model, represents a point-of-no-return. The phenotypic worsening could not be attributed to potential off-target effects – that were not examined – as an earlier AAV.U6-shNat8l.1-MBP-ASPA delivery was tolerated (not shown).

Our data have identified an age limit for a beneficial treatment outcome in the AKO mouse model to be at least 3 months, which is equivalent to humans in their third decade (Dutta and Sengupta, 2016). This may suggest a potential late intervention option for CD patients with the milder juvenile type or even for some patients with the more severe infantile form who live into their late adolescence or adulthood (Mendes et al., 2017).

In the healthy CNS, the endogenous expression domain of ASPA is restricted to oligodendrocytes (Klugmann et al., 2003; Madhavarao et al., 2004; von Jonquieres et al., 2018). As such it appears intuitive to target AAV.ASPA gene delivery to this cell type. In fact, AAV-mediated *ASPA* gene augmentation in oligodendroglia phenotypically restored the CD-like pathology in two different symptomatic models (von Jonquieres et al., 2018; Francis et al., 2021). Separately, AAV-mediated *ASPA* gene delivery to neurons or astrocytes has also shown promising results in CD mouse models (Ahmed et al., 2013; Gessler et al., 2017). These findings indicate that promiscuous ASPA expression in cell types other than the original target will be tolerated and might even contribute to creating a metabolic sink for excess NAA.

Expanding on proof-of-concept studies that have shown the benefits of ASPA expression broadly in the CNS, two clinical AAV.ASPA trials are currently in Phase I/II (NCT04998396 and NCT04833907). Non-ASPA targets were identified in preclinical gene therapy settings or gene knockout experiments and included NAT8L suppression (Bannerman et al., 2018; Hull et al., 2020) or inhibiting the NAA transporter NAC3 (Wang et al., 2021). Our data suggest that the latter target could be enabled by our dual-function vector approach.

Taken together, this study showed feasibility data for a dual-function vector-based gene therapy approach in late-stage CD mice. The substantial treatment effects across all vectors examined resulted in reversal of the pathology that was present at the time of intervention. These strong and uniform effects with all vectors precluded a clear ranking of the candidate AAV-cassette designs for biopotency. In order to exploit the full potential of the dual-function design, optimizations will address more promoters, the relative orientation of the two cistrons, spacers, and other genetic elements. The dual-function vectors developed and tested in this study are a powerful tool for post-symptomatic CD gene therapy with the prospect to revert already established CD pathology late into disease progression.

## Data availability statement

The raw data supporting the conclusions of this article will be made available by the authors, without undue reservation.

## Ethics statement

The animal study was reviewed and approved by University of New South Wales Animal Care and Ethics Committee.

## Author contributions

DF and MK designed the study and led the project and manuscript preparation. DF, EK, AB, BL, AKS, and MK conducted the research. DF, EK, AB, BL, and MK analyzed the data. GvJ and GDH contributed to the experimental design and manuscript preparation. All authors contributed to the article and approved the submitted version.

## Funding

This work was funded by the European Leukodystrophy Association (ELA 2015-017I1) and the Australian Government Medical Research Future Fund (Leukodystrophy Flagship – Massimo’s Mission; MRFF-ARLKO).

## Conflict of interest

MK was employed by the company Boehringer Ingelheim Pharma GmbH & Co. KG.

The remaining authors declare that the research was conducted in the absence of any commercial or financial relationships that could be construed as a potential conflict of interest.

## Publisher’s note

All claims expressed in this article are solely those of the authors and do not necessarily represent those of their affiliated organizations, or those of the publisher, the editors and the reviewers. Any product that may be evaluated in this article, or claim that may be made by its manufacturer, is not guaranteed or endorsed by the publisher.

## Footnotes

^1^ http://sirna.wi.mit.edu
